# Supplementary material for: Exploring Triaging and Short-Term Outcomes of Early Invasive Strategy in Non-ST Segment Elevation Acute Coronary Syndrome: A Report from Japanese Multicenter Registry
Source: J Clin Med. 2020 Apr 13;9(4):1106. doi: 10.3390/jcm9041106 (PMC7230808; doi:10.3390/jcm9041106)
Supplement: Supplementary file 1 [file jcm-09-01106-s001.pdf]

Supplementary materials: Exploring Triaging and Short-Term Outcomes of Early Invasive Strategy in Non-ST Segment Elevation Acute Coronary Syndrome: A Report from Japanese Multicenter Registry

Nobuhiro Ikemura, Yasuyuki Shiraishi, Mitsuaki Sawano, Ikuko Ueda, Yohei Numasawa, Shigetaka Noma, Masahiro Suzuki, Yukihiko Momiyama, Kentaro Hayashida, Shinsuke Yuasa, Hiroaki Miyata, Keiichi Fukuda and Shun Kohsaka

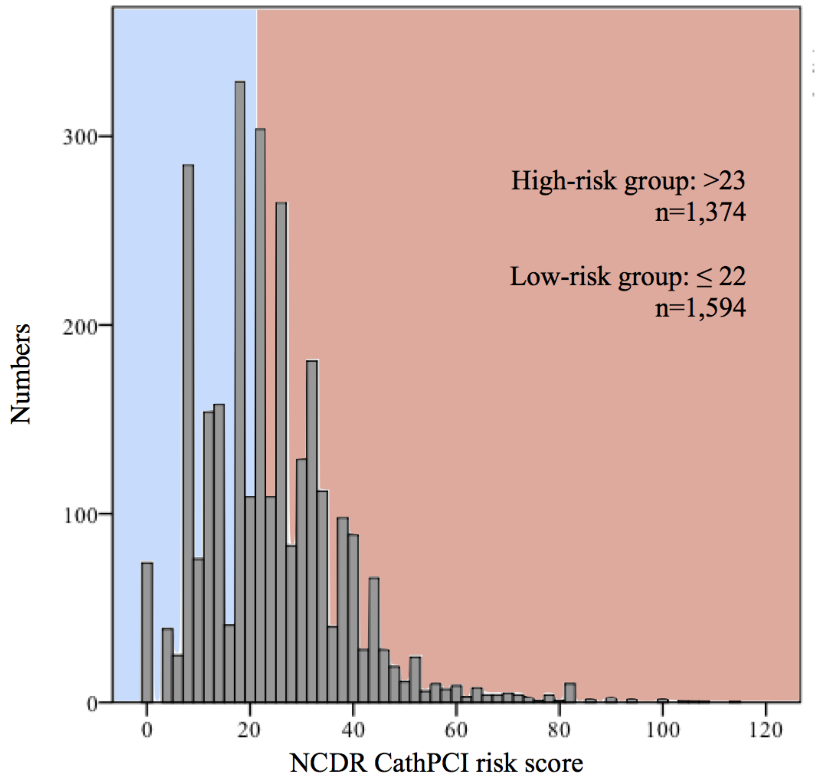

Figure S1. Overall distribution of the National Cardiovascular Data Registry (NCDR) CathPCI risk score.

Table S1. Baseline characteristics between patients with and without AKI.

Variable	Patients without AKI (n=2,289)		Patients with AKI (n=375)		P value
	numbers	%	numbers	%	
Male	1823	79.6	262	69.9	<0.001
Age, median (Q1-Q3)	68 (60–76)		75 (65–80)		<0.001
BMI, median (Q1-Q3)	23.9 (21.8–26.3)		23.7 (21.3–25.7)		0.91
Family history of CAD	313	13.7	33	8.9	0.011
Current smoker	790	34.5	134	35.8	0.62
Past medical history					
Hypertension	1719	75.1	294	78.4	0.16
Diabetes mellitus	851	37.2	163	43.6	0.018
Dyslipidemia	1529	66.8	249	66.4	0.88
CKD	805	35.2	166	44.3	<0.001

eGFR, median (Q1-Q3)	71.7 (54.3–94.7)		60.3 (37.3–93.5)		0.011
PAD	137	6.0	21	5.6	0.77
COPD	80	3.5	14	3.7	0.81
Prior heart failure	109	4.8	39	10.4	<0.001
Prior MI	413	18.0	64	17.1	0.64
Prior PCI/CABG	686	30.0	82	21.9	0.001
Situation at arrival					
NSTEMI	1003	43.8	230	61.3	<0.001
Unstable angina	1286	56.2	145	38.7	<0.001
Heart failure	207	9.0	85	22.7	<0.001
NYHA 3 or 4	118	5.2	57	15.2	<0.001
EF, median (Q1-Q3)	63 (55–70)		60 (45–67)		<0.001
EF <40%	92	4.0	34	9.1	<0.001
Cardiogenic shock	47	2.1	24	6.4	<0.001
CPA	37	1.6	15	4.0	0.002
Elevated cardiac troponin	729	31.8	180	48.0	<0.001
NCDR CathPC risk score, median (Q1-Q3)	22 (14–30)		28 (20–39)		<0.001
NCDR CathPCI risk score, mean, (SD)	24.3 (14.1)		21.9 (12.7)		
Procedure characteristics					
Femoral approach	1161	50.8	249	66.6	<0.001
Use of IABP	111	4.8	63	16.8	<0.001
Use of VA-ECMO	9	0.4	8	2.1	<0.001
LMT lesion	85	3.7	20	5.3	0.13
LAD lesion	1178	51.5	198	52.8	0.63
LCX lesion	581	25.4	128	34.1	<0.001
RCA lesion	660	28.8	108	28.8	0.98
Multivessel PCI	211	9.2	70	18.7	<0.001
Fluoroscopy time, min, median, (Q1-Q3)	23.4 (15.5–36.0)		27.0 (18.2–41.0)		<0.001
Contrast volume, ml median, (Q1-Q3)	156 (125–200)		170 (125–220)		0.034

Abbreviations: EIS, early invasive strategy; PCI, percutaneous coronary intervention; BMI, body mass index; CAD, coronary arterial disease; CKD, chronic kidney disease (eGFR<60 ml/min/1.73m^2); eGFR, estimated glomerular filtration rate; HD, hemodialysis; MI, myocardial infarction; PAD, peripheral artery disease; COPD, chronic obstructive pulmonary disease; CABG, coronary artery bypass grafting; NSTEMI, non-ST elevation myocardial infarction; NYHA, New York Heart Association classification; EF, ejection fraction; CPA, cardiopulmonary arrest; NCDR, National Cardiovascular Data Registry; IABP, Intra-aortic balloon pump; VA-ECMO, veno-arterial Extracorporeal membrane oxygenation; LMT, left main trunk; LAD, left anterior descending; LCX, left circumflex; RCA, right coronary artery; Q, quartile.

Table S2. The results of logistic regression models evaluating whether an early invasive strategy was independently associated with an in-hospital mortality.

	**Adjusted OR**	95% confidence interval		P value
		Lower limit	Upper limit	
Early invasive strategy	**0.99**	0.43	2.29	0.98
Male (vs female)	0.83	0.42	1.65	0.60
Age (≥75 years old)	1.45	0.69	3.04	0.33
Prior heart failure	2.70	1.18	6.13	0.02
Diabetes mellitus	1.28	0.69	2.39	0.43
Peripheral artery disease	1.19	0.49	2.85	0.70
Chronic kidney disease*	4.07	1.78	9.28	<0.001
NYHA 3 or 4	1.53	0.60	3.92	0.37
Elevated cardiac troponin	2.75	1.46	5.17	0.00
Prior PCI/CABG	1.09	0.56	2.13	0.79

Cardiogenic shock/CPA	26.9	13.6	53.1	<0.001

*: estimated glomerular filtration rate <60 ml/min/1.73m^2. **Abbreviations:** OR, odds ratio; NYHA, New York Heart Association classification; PCI, percutaneous coronary intervention; CABG, coronary artery bypass grafting; CPA, cardiopulmonary arrest.

Table S3. The results of logistic regression models evaluating whether an early invasive strategy was independently associated with a bleeding complication after PCI.

	**Adjusted OR**	95% confidence interval		P value
		Lower limit	Upper limit	
Early invasive strategy	0.91	0.52	1.58	0.72
Male (vs female)	0.45	0.29	0.71	0.001
Age (≥75 years old)	0.93	0.58	1.49	0.76
Prior heart failure	1.89	1.08	3.33	0.026
Diabetes mellitus	1.12	0.73	1.72	0.59
Peripheral artery disease	0.80	0.38	1.72	0.57
Chronic kidney disease*	2.24	1.36	3.71	0.002
NYHA 3 or 4	2.39	1.34	4.26	0.003
Elevated cardiac troponin	1.65	1.08	2.50	0.019
Prior PCI/CABG	0.77	0.47	1.25	0.28
Cardiogenic shock/CPA	6.49	3.51	11.99	<0.001

*: estimated glomerular filtration rate <60 ml/min/1.73m^2. **Abbreviations:** OR, odds ratio; NYHA, New York Heart Association classification; PCI, percutaneous coronary intervention; CABG, coronary artery bypass grafting; CPA, cardiopulmonary arrest. **.**
